# Urban Flood Risk Assessment Based on Dynamic Population Distribution and Fuzzy Comprehensive Evaluation

**DOI:** 10.3390/ijerph192416406

**Published:** 2022-12-07

**Authors:** Hao Chen, Zongxue Xu, Yang Liu, Yixuan Huang, Fang Yang

**Affiliations:** 1 College of Water Sciences, Beijing Normal University, Beijing 100875, China; 2 Beijing Key Laboratory of Urban Hydrological Cycle and Sponge City Technology, Beijing 100875, China; 3 College of Geoscience and Surveying Engineering, China University of Mining and Technology, Beijing 100875, China; 4 The Pear River Hydraulic Research Institute, Pearl River Water Resources Commission, Guangzhou 510000, China

**Keywords:** urban flood risk assessment, dynamic population, improved entropy weight, fuzzy comprehensive evaluation, principle of maximum membership

## Abstract

Floods are one of the most common natural disasters that can cause considerable economic damage and loss of life in many regions of the world. Urban flood risk assessment is important for urban flood control, disaster reduction, and risk management. In this study, a novel approach for assessing urban flood risk was proposed based on the dynamic population distribution, improved entropy weight method, fuzzy comprehensive evaluation method, and the principle of maximum membership, and the spatial distribution of flood risk in four different sessions or daily time segments (TS1–TS4) in the northern part of the Shenzhen River Basin (China) was assessed using geographic information system technology. Results indicated that risk levels varied with population movement. The areas of highest risk were largest in TS1 and TS3, accounting for 7.03% and 7.07% of the total area, respectively. The areas of higher risk were largest in TS2 and TS4, accounting for 4.54% and 4.64% of the total area, respectively. The findings of this study could provide a theoretical basis for assessing urban flood risk management measures in Shenzhen (and even throughout China), and a scientific basis for development of disaster prevention and reduction strategies by flood control departments.

## 1. Introduction

Floods are one of the most common natural disasters and can cause considerable economic damage and loss of life in many regions of the world [1,2,3,4]. Previous studies reported that floods accounted for 44% of all disaster events during 2000–2019, affecting 1.6 billion people worldwide and causing economic losses of US$651 billion [5]. Moreover, with global climate change and acceleration of urbanization processes, the frequency and risk level of floods are expected to increase in the future [6,7].

During the past two decades, China has been affected most by flooding, experiencing an average of 20 floods per year that affected a total of 900 million people, i.e., approximately 55% of the total number of people affected by flooding worldwide [5]. Statistical data in China’s flood and drought disaster prevention bulletin reveal that during 2000–2020 there were 21,720 fatalities and economic losses of 3163.952 billion yuan directly attributable to flooding. Urban floods, which have become an important factor of urban public security in China, seriously restrict sustainable and healthy socioeconomic development [8]. As an important tool for flood prevention, flood risk assessment has substantial practical application in flood risk management. It not only provides scientific and technological support for urban flood prevention and disaster reduction, but also leads to improvement in public awareness of flood risk. Consequently, flood risk assessment has become a hot topic in the field of natural science and technology over recent decades [9,10,11].

Urban flood risk assessment performs comprehensive evaluation of hazard-formative factors, hazard-formative environments, hazard-affected bodies, and the capability for disaster prevention and reduction with the aim of obtaining accurate assessment of flood risk levels [12]. Traditionally, flood risk assessment adopts three main approaches: historical disaster mathematical statistics methods [13,14,15], multicriteria analysis (MCA) [16,17,18,19], and scenario simulation analysis [20,21,22,23]. Among these three approaches, MCA is used more widely in research on urban flood risk assessment because it can better reflect the chain of urban flood disasters, and has the advantages of easy data acquisition and simple modeling. However, related studies often use the average annual population distribution as one of the indicators for analysis when using MCA for urban flood risk assessment, thereby ignoring the dynamic changes of the population and reducing the accuracy of the assessment results.

Accurate information of the actual population distribution and its temporal patterns is of high importance for calculating populations at risk for human or natural disasters, to assess vulnerabilities, or to derive health and development indicators [24,25]. However, information about the actual human distribution is often scarce and predominantly based on static population data derived from national population censuses and registers. Although the increasing use of global positioning and geographic information system technologies has supported the improved collection of census data and their processing, these much-used datasets only provide information about the static population rather than the actual distribution of people at different times of the day [26,27]. In recent years, with the popularity of mobile phones, how to capture population dynamics in space and time has fundamentally changed, because they can reveal the precise digital footprints of individuals in space and time. Analyses of population dynamic distribution based on mobile phone data are of great significance for improving the accuracy of disaster risk assessment and the response to disasters [28,29]. However, to date, the applications of dynamic population distribution in urban flood risk assessment are still rare. Therefore, in this study, we integrated the dynamic population into MCA to develop a multi-index system of flood risk assessment.

The assignment of index weights in MCA is crucial to the result of its application to urban flood risk assessment [30]. Common methods to determine appropriate index weights include the analytic hierarchy process (AHP) [1,31,32,33] and the entropy method [34,35]. These two methods have their own specific advantages and disadvantages. For example, the AHP is greatly affected by the expert cognitive level of the user and it exhibits strong randomness. The entropy method is mainly affected by the characteristics of the data distribution, without considering the practical significance of the indexes [23,36,37]. Therefore, an improved entropy weight (EW) method that incorporates the advantages of both the AHP and the entropy method was adopted in this study.

We propose a novel approach for assessing urban flood risk based on the dynamic population distribution, improved EW method (combined weight method), fuzzy comprehensive evaluation method, and the principle of maximum membership, and we assess the spatial distribution of flood risk in the northern part of the Shenzhen River Basin (China) with the support of geographic information system (GIS) technology. The remainder of this paper is organized as follows. In Section 2, the study area and data are introduced. Section 3 describes the methods adopted in this study, including the combined weight method and fuzzy comprehensive evaluation. The results of the flood risk assessment based on the dynamic population distribution, combined weight method, fuzzy comprehensive evaluation method, and the principle of maximum membership in the study area are reported and discussed in Section 4. Finally, the derived conclusions are described in Section 5.

## 2. Study Area and Data Description

### 2.1. Study Area

**Figure 1:**
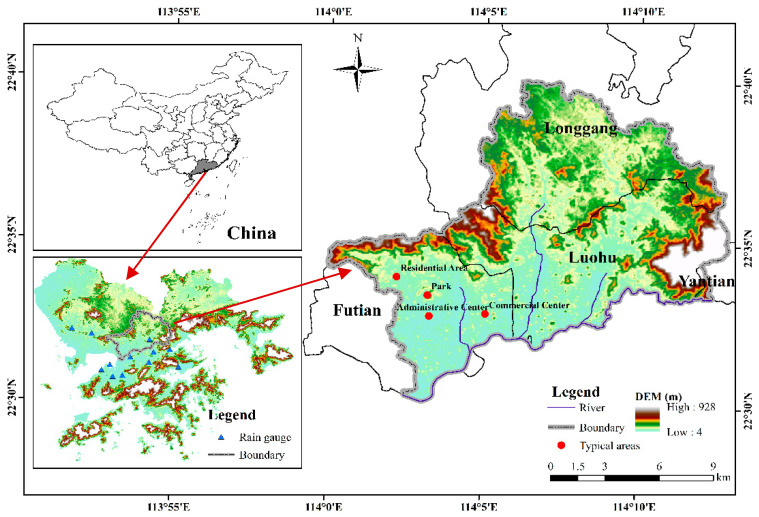
Location of the study area.

The Shenzhen River Basin is located to the east of the Pearl River Estuary in Southeast China, which is an important part of the Guangdong–Hong Kong–Macao Greater Bay Area. The catchment is bordered by mountains to the north, east, and south and by Shenzhen Bay to the west. The basin area to the north of the Shenzhen River belongs to the city of Shenzhen and the area to the south belongs to the Hong Kong SAR. The Shenzhen River originates at Niuweiling in the Wutong Mountains and flows southwestward over a distance of 33.4 km into Shenzhen Bay. Its major tributaries include the Shawan, Buji, and Futian rivers on the Shenzhen side, and the Pingyuan, Wutong, and Xintian rivers on the Hong Kong side. We selected the northern part of the Shenzhen River Basin (total area: 192.69 km^2^; Figure 1) as the study area for urban flood risk assessment based on the dynamic population distribution. The study area has a maritime subtropical monsoon climate with average annual precipitation of 1764.14 mm.

The study area, which includes central and eastern parts of Futian District, all of Luohu District, the southwest of Longgang District, and the western edge of Yantian District, represents the most economically developed and densely populated area of Shenzhen. Owing to its special geographical location, this area is vulnerable to joint impact from rainfall and tide level, which means urban floods occur frequently and seriously hinder the sustainable socioeconomic development of the region. On the evening of 11 April 2019, Shenzhen was hit by short-time extreme heavy rainfall, which led to sudden floods in several regions of the city, resulting in disaster in some areas and 11 deaths. All the dead were concentrated in Luohu and Futian District. From 29 to 30 August 2018, heavy rainstorms occurred in Shenzhen for two consecutive days, with the maximum 24-h rainfall reaching 413 mm. Different degrees of disasters have occurred in the whole city, with Luohu and Longgang District suffering the most. Furthermore, in the “5.11” (2014), “6.13” (2008) and other heavy rainstorm events in Shenzhen, the study area was the most severely affected. Therefore, it is very important that urban flood risk assessment be undertaken in this area.

### 2.2. Data Description

The selection of risk index varies by region and depends on the specific characteristics of the location [10]. One index could have a high degree of impact toward flood risk in one area but could be neglected in another area [38]. According to the actual conditions of hazard-formative factors, hazard-formative environments, hazard-affected bodies, and the capability for disaster prevention and reduction, starting from the two risk elements of hazard and vulnerability, this study selected 10 flood risk indexes for analysis: maximum 1-day rainfall amount (Rx1day), number of heavy rainfall days above 50 mm (R50mm), digital elevation model (DEM), slope (SL), land use pattern (LUP), distance to the river (DR), gross domestic product density (GDP), dynamic population (D-POP), building density (BD), and pipe network density (PD). Each index is described in detail in the following.

- Maximum 1-day rainfall amount (Rx1day, mm). Precipitation is a direct factor leading to urban flooding. Rx1day reflects the extreme precipitation leading to floods in the study area. The Rx1day index was calculated according to daily precipitation data recorded at observational stations during 1986–2019. The data were obtained from Shenzhen hydrological data almanacs and the University of Hong Kong.
- Number of heavy rainfall days above 50 mm (R50 mm, number/year). R50 mm reflects the frequency of extreme precipitation. The length and source of the data series were consistent with those of Rx1day.
- Digital elevation model (DEM, m). This index reflects the topographical conditions of the study area. Areas at low elevation are generally more prone to flooding because rainfall flows from areas of high elevation to areas of low elevation under natural conditions. The DEM data were accessed from the Geospatial Data Cloud (https://www.gscloud.cn/, accessed on 20 May 2020).
- Slope (SL, degree). This index is used to reflect the degree of topographic change. Mountain areas generally have severe slopes that prevent water collection, whereas lowlands or flatlands have gentle slopes that result in a constant threat of flooding. The index was calculated from the DEM using GIS techniques.
- Land use pattern (LUP). Runoff conditions vary considerably under different patterns of land use. A runoff coefficient was used to differentiate the LUP. Seven patterns were selected and assigned different runoff coefficients according to the Code for the Design of Building Water Supply and Drainage of China (GB50015-2003) and the Code for the Design of Outdoor Wastewater Engineering of China (GB50014-2006). The runoff coefficient of forest land, herbaceous land, wet land, crop land, bare area, built-up area, and water bodies was set as 0.15, 0.2, 0.5, 0.6, 0.7, 0.9, and 1, respectively. The land use data were accessed from the European Space Agency (https://viewer.esa-worldcover.org/worldcover, accessed on 20 May 2020).
- Distance to the river (DR, km). Regions near rivers might be prone to flooding because of dyke breaching or overtopping. Conversely, regions far away from rivers are generally safer. The DR index was set to 0 at the river and its value increased as the distance to the river increased. This step was conducted using Euclidean distance in the GIS.
- Gross domestic product density (GDP, yuan/km^2^). This index is important for reflecting the economic situation and the level of social development of the study area in 2019. The data were derived from a geographic remote sensing ecological network.
- Dynamic population (D-POP, people/km^2^). Population distribution is crucial regarding the accuracy of the results of urban flood risk assessment. In this study, D-POP reflected the dynamic change of population within the study area. The data were accessed based on location information clustering of Baidu products (e.g., web search, maps, weather, and music) accessed by smartphones.
- Building density (BD). The index refers to the ratio of the building area in each grid to the total grid area within the study area. The data were accessed from BIGMAP.
- Pipe network density (PD, km/km^2^). The index refers to the length of the drainage pipe network in each grid, which reflects the capability of urban flood control and disaster reduction to a certain extent. The drainage pipe network data were provided by the China Institute of Water Resources and Hydropower Research.

Owing to the different treatment of positive and negative indexes, and according to the above description and analysis of each index, the indexes were divided into positive (Rx1day, R50mm, LUP, GDP, D-POP, and BD) and negative (DEM, SL, DR, and PD) indicators (Table 1). The greater the value of positive indicators, the higher the risk. Negative indicators were the opposite.

## 3. Method

**Figure 2:**
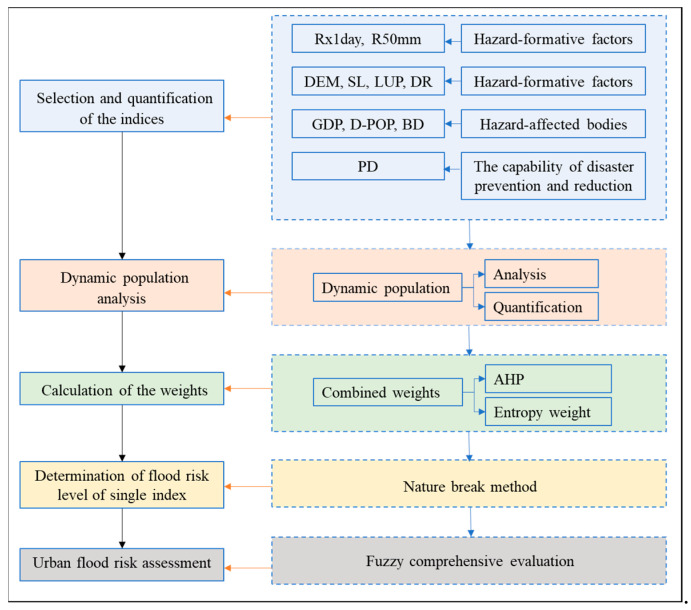
Overall framework and procedure of urban flood risk assessment.

The overall framework and procedure of urban flood risk assessment are shown in Figure 2. The approaches used in this study involve two main components: weight calculation and fuzzy comprehensive evaluation. Each of these two approaches is described in detail in the following.

### 3.1. Calculation of the Weights for the Indexes

#### 3.1.1. Analytic Hierarchy Process (AHP)

The AHP is a multicriteria decision-making method for assessing and integrating the impacts of various factors [39]. It can make full use of the knowledge and experience of experts, and can explore the inherent laws of data to guarantee the rationality of the results and to reduce randomness. It includes four steps: establishment of a hierarchical model, construction of a judgment matrix, calculation of the weight of each layer, and a consistency check [40]. In this study, the AHP was used as a method to determine the subjective weights of the indexes.

#### 3.1.2. Entropy Weight (EW)

Entropy is a measure of the degree of disorder of information of a specific system and it can be used to fully exploit the information contained in original data [41]. The EW can indicate useful information provided by an index; however, it relies excessively on objective data that cannot reflect the knowledge and practical experience of experts, such that the results are occasionally inconsistent with reality and individual understanding. In recent years, the EW method has been used widely to calculate index weights. In this study, EW was used to determine the objective weights of the indexes. The main steps of the EW method are summarized in the following.

Step 1: An initial sample evaluation matrix *X* was constructed for *m* evaluated objects and *n* evaluation indexes:

(1) $$X=\left[\begin{array}{cccc} x_{11} & x_{12} & \cdots & x_{1n}\\ x_{21} & x_{22} & \cdots & x_{2n}\\ \vdots & \vdots & \ddots & \vdots \\ x_{m1} & x_{m2} & \cdots & x_{mn} \end{array}\right]$$

Step 2: Because the units of measurement of the various indexes are not unified, they must be normalized before being used to calculate comprehensive indexes, thereby overcoming the problem of homogenization of various heterogeneous index values. Moreover, because the values of the positive and negative indexes represented different meanings, we used different algorithms for data normalization of the positive and negative indexes. Therefore, sample matrix *X* was converted into normalized matrix *Y* as follows:

(a) normalize the positive indexes:

(2) $$y_{ij}=\frac{x_{ij}-\min(x_{ij})}{\max(x_{ij})-\min(x_{ij})}$$

(b) normalize the negative indexes:

(3) $$y_{ij}=\frac{\max(x_{ij})-x_{ij}}{\max(x_{ij})-\min(x_{ij})}$$

where $y_{ij}$ is the normalized value of the *i-th* sample of the *j-th* index (*I* = 1, 2,…, *m*; *j* = 1, 2,…, *n*).

Step 3: The entropy of the *j-th* index was defined as follows:

(4) $$\mathrm{let}\;p_{ij}=y_{ij}/\sum_{i=1}^{m}y_{ij}$$

(5) $$\mathrm{then},\;e_{j}=(-\sum_{i=1}^{m}p_{ij}\ln p_{ij})/\ln m$$

Step 4: The deviation degree $D_{j}$ of the *j-th* index was calculated as follows:

(6) $$D_{j}=1-e_{j}$$

Step 5: Finally, the weight of the *j-th* index was calculated as follows:

(7) $$w_{j}^{\prime }=D_{j}/(\sum_{j=1}^{n}D_{j})$$

#### 3.1.3. Combined Weights

The combined weight method, which integrates the advantages of the AHP and EW methods, can make full use of the knowledge and experience of experts, and can explore the inherent laws of the data to guarantee the rationality of the results and to reduce randomness. In this study, we used the AHP method to determine the subjective weight $w_{j}^{*}$ of the index, and the EW method to determine the objective weight $w_{j}^{\prime }$ of the index. The combined weight $w_{j}$ of the *j-th* index was calculated as follows:

(8) $$w_{j}=\left(w_{j}^{*}w_{j}^{\prime }\right)/(\sum_{j=1}^{n}w_{j}^{*}w_{j}^{\prime })$$

### 3.2. Fuzzy Comprehensive Evaluation

A fuzzy set can quantify the fuzziness of an object and make a reasonable evaluation through the membership function [42]. In comparison with traditional evaluation methods, the concepts and methods of fuzzy mathematics are more suitable for risks with fuzzy characteristics. Therefore, using fuzzy mathematics to establish a fuzzy evaluation model of flood risk is more in accord with the actual situation in comparison with using traditional evaluation methods. The basic steps of the procedure are described in the following.

Step 1: The combined weights of each index were calculated according to Equation (8), and the weight matrix W was constructed as follows:

(9) $$W=(w_{1},w_{2},\cdots ,w_{n})$$

Step 2: Each index was divided into five levels, and a comment set $V=(v_{1},v_{2},v_{3},v_{4},v_{5})$ was established, corresponding to the lowest risk, lower risk, medium risk, higher risk, and highest risk respectively. The upper limit value of each index was the maximum value and the lower limit value was the minimum value. Five segmentation points $\left(a_{1},a_{2},a_{3},a_{4},a_{5}\right)$ were selected for each index using the natural break method.

Step 3: Membership function is an important part of the fuzzy comprehensive evaluation method, which directly affects the accuracy of the evaluation results. In this study, the descending half trapezoid, ascending half trapezoid, and triangular distribution functions were selected to calculate the membership degree of each index to each risk level in the evaluation set. If $r_{jk}$ represented the membership degree of the *j-th* index to risk level *k*, the membership function could be expressed as follows:

(10) $$r_{j1}=\{\begin{array}{c} 1(x\leq a_{1})\\ \frac{a_{2}-x}{a_{2}-a_{1}}(a_{1}<x\leq \\ 0(x>a_{2}) \end{array}a_{2})$$

(11) $$r_{jk}=\{\begin{array}{c} \frac{x-a_{k-1}}{a_{k}-a_{k-1}}(a_{k-1}<x\leq a_{k})\\ \frac{a_{k+1}-x}{a_{k+1}-a_{k}}(a_{k}<x\leq a_{k+1})\\ 0(x>a_{k+1}\;or\;x\leq a_{k-1}) \end{array}$$

(12) $$r_{j5}=\{\begin{array}{c} 0(x\leq a_{4})\\ \frac{x-a_{4}}{a_{5}-a_{4}}(a_{4}<x\leq a_{5})\\ 1(x>a_{5}) \end{array}$$

where x is the actual data of an evaluation index, and $a_{k}$ is the critical value of the corresponding risk level. According to the membership functions, the fuzzy relationship matrix R between sample matrix *X* and comment set *V* was obtained.

Step 4: Using the algorithm of fuzzy mathematics, the weight matrix *W* and fuzzy relationship matrix R were calculated to obtain the composite fuzzy vector *B*:

(13) $$B=W\cdot R=(w_{1},w_{2},\cdots ,w_{10})\cdot \left[\begin{array}{cccc} r_{11} & r_{12} & \cdots & r_{15}\\ r_{21} & r_{22} & \cdots & r_{25}\\ \vdots & \vdots & \ddots & \vdots \\ r_{101} & r_{102} & \cdots & r_{105} \end{array}\right]=(b_{1},b_{2},b_{3},b_{4},b_{5})$$

### 3.3. Determination of Flood Risk Level

According to the principle of maximum membership degree, the fuzzy comprehensive evaluation vector B was compared, and the maximum value was selected as the basis for evaluation of flood risk level. For example, in the evaluation vector of an evaluation unit, $\max(b_{1},b_{2},b_{3},b_{4},b_{5})=b_{4}$ indicates that the risk level of the evaluation unit was the higher risk.

## 4. Results and Discussion

### 4.1. Quantification of the Indexes

**Figure 3:**
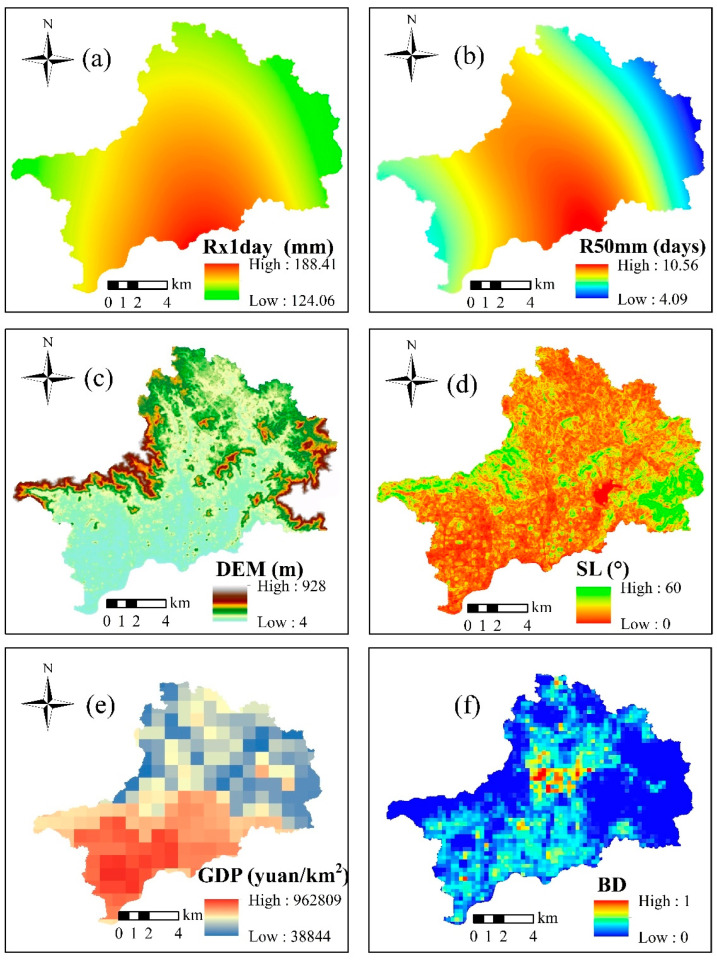

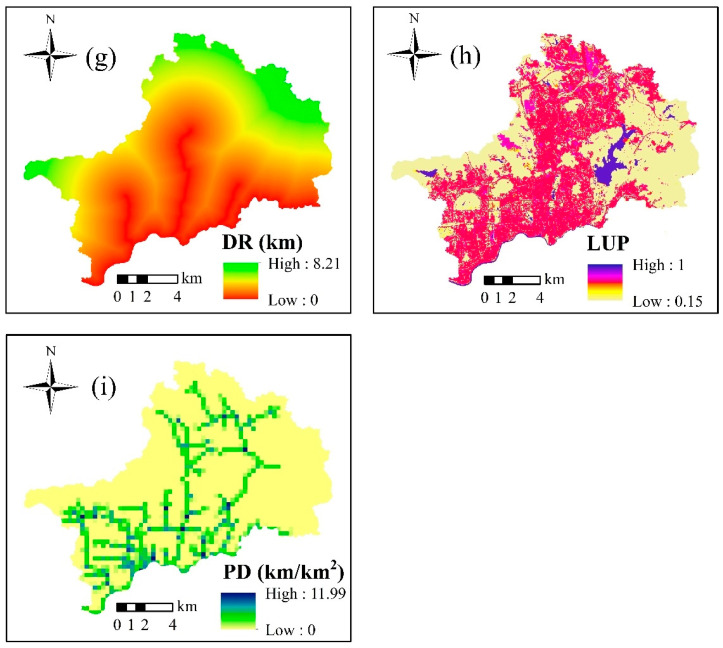
Spatial distributions of risk indexes: (**a**) Rx1day, (**b**) R50mm, (**c**) DEM, (**d**) SL, (**e**) LUP, (**f**) DR, (**g**) GDP, (**h**) BD, and (**i**) PD.

Based on the principles of being scientific, systematic, quantitative (measurable), operational, and universal, the indexes of Rx1day, R50mm, DEM, SL, LUP, DR, GDP, D-POP, BD, and PD were selected to construct a flood risk assessment system for the northern part of Shenzhen River Basin. Except for the D-POP index, the other indicators were transformed into grid layers by GIS wherein the raster scale was set to 30 × 30 m, and the study area was divided into 214,280 grids, as shown in Figure 3.

### 4.2. Dynamic Population Analysis

In the process of urbanization, the number of super large cities with highly dense population is increasing, and population mobility is becoming more complex and diverse. Based on the average annual population distribution, urban flood risk assessment has gradually been unable to satisfy the requirements of urban flood prevention and disaster reduction. Establishment of an urban flood risk assessment system based on the dynamic population distribution is an inevitable requirement of urban development. In this study, we obtained the dynamic population distribution of Shenzhen from 20 April 2020 to 20 May 2020 based on the location information clustering of Baidu products accessed by smartphones, with 1-h temporal resolution and 200-m spatial resolution. The dynamic population of the study area was analyzed based on a GIS platform.

**Figure 4:**
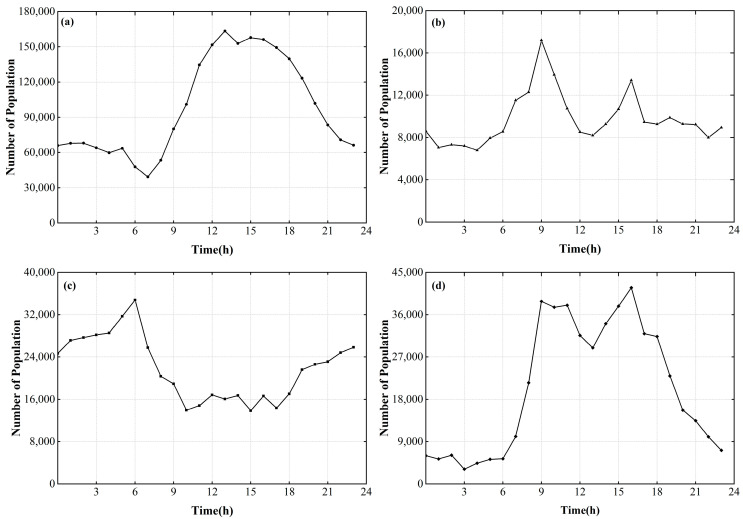
Dynamic changes in population of typical areas of Shenzhen: (**a**) commercial center, (**b**) park, (**c**) residential area, and (**d**) administrative center.

We selected four typical areas (Figure 1), including a commercial center, park, residential area, and administrative center, to analyze the daily dynamic changes in population, as shown in Figure 4.

(1) Commercial center. Figure 4a shows the dynamic changes in population of the Shenzhen Huaqiang North Commercial Area (1.44 km^2^), which is the most densely populated area in Shenzhen. The population began to increase at 09:00 local time (LT) and reached a peak at around 13:00 LT. After 22:00 LT, the population was broadly stable at 60,000.

(2) Park. Figure 4b shows the dynamic changes in population of Lotus Hill Park. The main feature of the population change in this area is the two peaks that formed at 09:00 and 14:00 LT.

(3) Residential area. Figure 4c shows the dynamic changes in population of the Lower Merlin community. This area (0.47 km^2^) is dominated by residential areas and supporting commercial areas. The population began to decline from 06:00 LT, fluctuated slightly during 09:00–16:00 LT, and gradually increased after 17:00 LT.

(4) Administrative center. Figure 4d shows the dynamic changes in population of the Shenzhen Civil center. The area (0.14 km^2^) is dominated by public services such as municipal government administrative offices and the library. The population of this area began to increase from 06:00 LT, fluctuated during 09:00–16:00 LT, and decreased rapidly after 17:00 LT.

**Figure 5:**
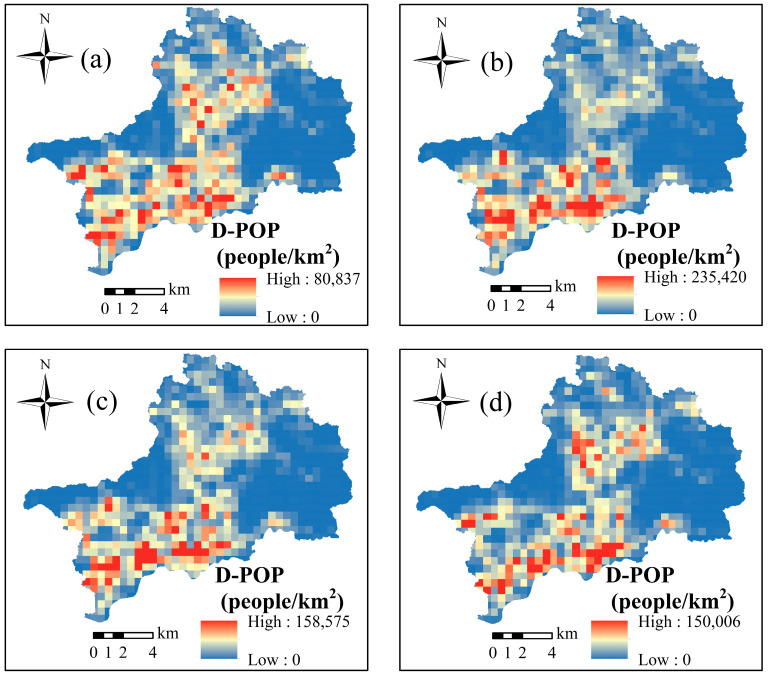
Average population distribution within the study area in different sessions: (**a**) TS1 (06:00–08:00 LT), (**b**) TS2 (09:00–16:00 LT), (**c**) TS3 (17:00–22:00 LT), and (**d**) TS4 (23:00–05:00 LT).

Based on the daily dynamic changes in population of the typical areas of Shenzhen, we divided a day into four sessions or time segments: TS1 (06:00–08:00 LT), TS2 (09:00–16:00 LT), TS3 (17:00–22:00 LT), and TS4 (23:00–05:00 LT), and we calculated the hourly average population of each session based on the GIS platform. Similarly, the results were converted into grid layers based on the GIS platform, and the raster scale was also set to 30 × 30 m. The results are shown in Figure 5.

As can be seen from Figure 5, because TS1 covered the morning rush hour, the population was most dispersed, and the maximum population density was only 80,837 people/km^2^. The population distribution of TS2 was the most concentrated, and the maximum population density reached 235,420 people/km^2^. The population was concentrated mainly in the most economically developed areas in the southwest of the study area, including the east of Futian District and the west of Luohu District. TS3 covered the late rush hour, and the population gathered in the southwest of the study area gradually migrated toward the north. TS4 covered the nighttime period during which the population was concentrated mainly in the residential areas of Futian District, Luohu District, and Longgang District.

### 4.3. Calculation of the Weights

As described in Section 3.1, the index weights were calculated using the combined weight method that integrates the advantages of the AHP and EW methods. First, the AHP method was applied to determine the relative degree of importance between the indexes. We invited 10 experts in the field of urban flooding and asked them to rank the significance of the indexes in terms of flood risk. Based on the significance assigned by the experts to the indexes in terms of flood risk, the judgment matrices of the indexes were determined (Table 2), with a consistency ratio CR = 0.021 < 0.1, thereby indicating that the judgment matrix was reasonable. Subsequently, the EW method was used to determine the objective weights based on the deviation degree within the index in different sessions. Table 3 provides details of the inconsistency between the entropy weights and the AHP weights. For example, DEM was considered an important index for flood risk estimation in the AHP method, whereas its weight was relatively low in the entropy method owing to the flat terrain in the study area and the small variation in elevation. Therefore, it was considered unreasonable to determine weights using only the AHP method or the EW method. The final index weights of the different sessions determined by the combined weight method are listed in Table 3.

### 4.4. Urban Flood Risk Assessment

**Figure 6:**
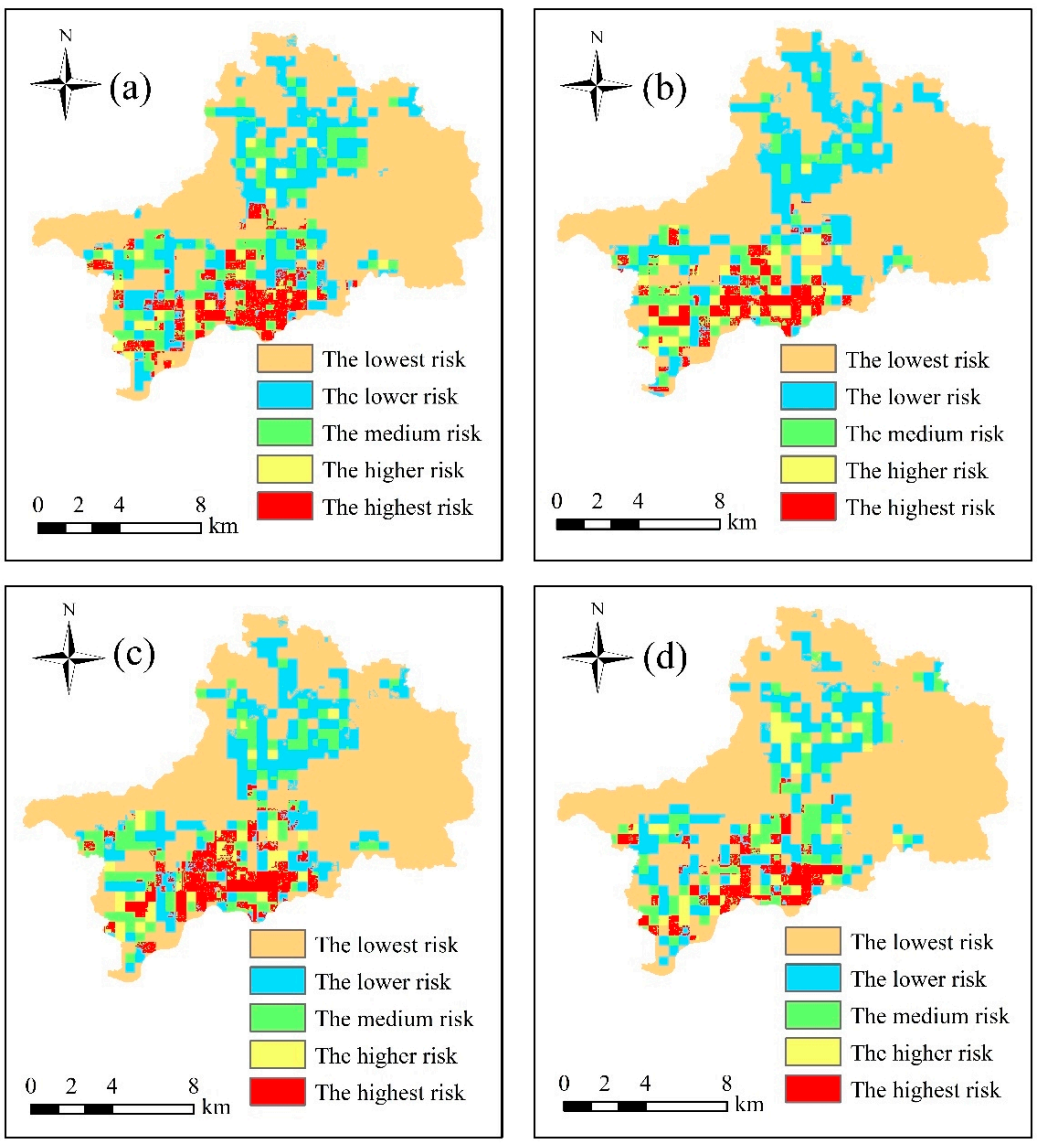
Maps of the spatial distribution of flood risk level in different sessions in the study area based on the fuzzy comprehensive evaluation: (**a**) TS1 (06:00–08:00 LT), (**b**) TS2 (09:00–16:00 LT), (**c**) TS3 (17:00–22:00 LT), and (**d**) TS4 (23:00–05:00 LT).

In this study, we assessed the urban flood risk of the study area based on the combined weight method, fuzzy comprehensive evaluation method, and the principle of maximum membership. The upper limit values of the nine indexes (Rx1day, R50mm, DEM, SL, LUP, DR, GDP, BD, and PD) were the maximum values, and the lower limit values were the minimum values. Because the maximum value of the D-POP index in the different sessions was different, the maximum value of the D-POP index in the four sessions was selected as the upper limit. Five segmentation points were selected for each index using the natural break method. The results are shown in Table 4. The calculation results obtained using the combined weights method for the different sessions are shown in Table 3. Finally, maps of the spatial distribution of flood risk level in the different sessions in the study area based on the fuzzy comprehensive evaluation were prepared, as shown in Figure 6.

It can be seen from Figure 6 that the areas with the highest flood risk level are concentrated mainly in the east of Futian District and the southwest of Luohu District. In TS1, the areas with the highest risk of urban flooding are concentrated mainly in the port area of Luohu District, owing to the large size of the outbound and traveling population gathered in this area during the morning rush hour, the relatively closeness of this area to the Shenzhen River, and the higher level of economic development. The flood risk level in Longgang District is mainly medium and low. During TS2, parts of the Luohu port area with the highest risk transform into high- and medium-risk areas, and the areas of highest risk decrease. Similarly, parts of the medium-risk areas in Longgang District transform into areas with lower risk. During TS3, the areas with highest risk gradually spread northward. Parts of Longgang District with lower risk transform into medium-risk areas. During TS4, the areas of highest risk in the east of Futian District and the southwest of Luohu District decrease, and some areas of Longgang District transform from medium-risk areas to higher-risk areas.

For the different sessions, the proportions of the areas with different risk levels to the total study area are listed in Table 5. It can be seen that the areas of highest risk in TS1 and TS3 were largest, accounting for 7.03% and 7.07% of the total area of the study area, respectively. This was because TS1 and TS3 covered the morning and late rush hours when the population was relatively scattered. The areas of higher risk in TS2 and TS4 were largest, accounting for 4.54% and 4.64% of the total area of the study area, respectively. This was because people were mainly in office areas and residential areas in TS2 and TS4, i.e., the population was relatively concentrated, which caused parts of the areas of highest risk to transform into areas of higher risk.

## 5. Conclusions

The study proposed a novel approach for assessing urban flood risk based on the dynamic population distribution, improved EW method (combined weight method), fuzzy comprehensive evaluation method, and the principle of maximum membership. With the support of GIS technology, the proposed approach was applied to assess the spatial distribution of the urban flood risk in different sessions in the northern part of the Shenzhen River Basin. Ten flood risk indexes (i.e., Rx1day, R50mm, DEM, SL, LUP, DR, GDP, D-POP, BD, and PD) were selected to construct the index system of urban flood risk assessment based on the principles of being scientific, systematic, quantitative (measurable), operational, and universal.

The assignment of index weights is crucial to the results of urban flood risk assessment. The AHP and EW methods were used to calculate subjective and objective weights of the indexes, and the results demonstrated inconsistency between the weights of the EW method and those of the AHP method. Therefore, it was considered unreasonable to determine index weights using only subjective methods or objective methods. In this study, we calculated the index weights based on a combined weight method that incorporates the advantages of both the AHP and the EW methods.

Finally, maps of the spatial distribution of flood risk level in different sessions were developed for the study area based on the dynamic population distribution, combined weight method, fuzzy comprehensive evaluation method, and the principle of maximum membership. The study results indicated that the highest risk areas were concentrated mainly in the east of Futian District and the southwest of Luohu District. The areas of highest risk were largest in TS1 and TS3, accounting for 7.03% and 7.07% of the total area of the study area, respectively. The areas of higher risk were largest in TS2 and TS4, accounting for 4.54% and 4.64% of the total area of the study area, respectively.

The study developed a new method for urban flood risk assessment based on the dynamic population distribution, which is expected to provide a theoretical basis for assessing urban flood risk management measures in Shenzhen (and even throughout China), and a scientific basis for development of disaster prevention and reduction strategies by flood control departments. In the future, with further developments of big data and other technologies, e.g., real-time traffic data and commuter travel data, a more comprehensive index system and more accurate urban flood risk assessment results could be obtained.

## Figures and Tables

**Table 1:** Urban flood risk assessment indexes and their attributes.

Schemes	Criteria	Indices	Units	Attributes of Indices
Hazard	Hazard-formative factors	Rx1day	mm	+
		R50mm	number/year	+
	Hazard-formative environment	DEM	m	-
		SL	degree	-
		LUP	/	+
		DR	km	-
Vulnerability	Hazard-affected bodies	GDP	yuan/km^2^	+
		D-POP	people/km^2^	+
		BD	/	+
	The capability of disaster prevention and reduction	PD	km/km^2^	-

**Table 2:** Judgment matrix for the AHP method.

Indices	Rx1day	R50mm	DEM	SL	LUP	DR	GDP	D-POP	BD	PD
Rx1day	1	4	5	7	6	5	3	2	7	2
R50mm		1	2	4	3	2	1/2	1/3	4	1/3
DEM			1	3	2	1	1/3	1/4	3	1/4
SL				1	1/2	1/3	0.2	1/6	1	1/6
LUP					1	1/2	1/4	1/5	2	1/5
DR						1	1/3	1/4	3	1/4
GDP							1	1/2	5	1/2
D-POP								1	6	1
BD									1	1/6
PD										1

**Table 3:** Determination of index weights based on the AHP, EW, and combined weight methods.

Indices	Methods								
	AHP	EW				Combined Weights			
		TS1	TS2	TS3	TS4	TS1	TS2	TS3	TS4
Rx1day	0.2596	0.0292	0.0286	0.0286	0.0283	0.0855	0.0816	0.0821	0.0801
R50mm	0.0808	0.0223	0.0217	0.0218	0.0215	0.0203	0.0193	0.0194	0.0190
DEM	0.0533	0.0033	0.0032	0.0032	0.0032	0.0020	0.0019	0.0019	0.0018
SL	0.0237	0.0066	0.0065	0.0065	0.0064	0.0018	0.0017	0.0017	0.0017
LUP	0.0352	0.2584	0.2523	0.2530	0.2499	0.1023	0.0977	0.0982	0.0959
DR	0.0533	0.0295	0.0288	0.0288	0.0285	0.0177	0.0169	0.0170	0.0166
GDP	0.1189	0.1591	0.1554	0.1558	0.1539	0.2129	0.2033	0.2044	0.1996
D-POP	0.1759	0.2432	0.2609	0.2588	0.2680	0.4816	0.5051	0.5024	0.5141
BD	0.0237	0.2428	0.2371	0.2378	0.2349	0.0648	0.0619	0.0622	0.0607
PD	0.1759	0.0057	0.0055	0.0056	0.0055	0.0112	0.0107	0.0108	0.0105

**Table 4:** Segmentation points of evaluation grade of each index.

Indices	$a_{1}$	$a_{2}$	$a_{3}$	$a_{4}$	$a_{5}$	Range of Indices
Rx1day	140.46	150.31	158.63	166.71	176.05	124.06–188.41
R50mm	5.92	7.04	7.97	8.84	9.65	4.09–10.56
DEM	522.00	335.00	196.00	105.00	48.00	4–928
SLOP	34.00	26.00	19.00	12.00	5.00	0–60
LUP	0.20	0.50	0.60	0.70	0.90	0.15–1
DR	5.99	4.60	3.32	2.09	0.97	0–8.21
GDP	83,665.00	182,638.00	267,973.00	404,129.00	680,763.00	38,844–962,809
POP	7385.71	20,310.71	36,005.34	58,162.48	110,785.67	0–235,420
BD	0.07	0.18	0.30	0.44	0.64	0–1
PD	7.52	5.41	3.81	2.30	0.71	0–11.99

**Table 5:** Areal proportions of different risk levels in different sessions in the study area (%).

Sessions	Risk Levels				
	Lowest Risk	Lower Risk	Medium Risk	Higher Risk	Highest Risk
TS1	58.32	19.47	12.00	3.17	7.03
TS2	59.32	21.28	8.88	4.54	5.99
TS3	59.61	18.88	10.66	3.78	7.07
TS4	62.97	17.16	10.00	4.64	5.22

## Data Availability

Not applicable.
